# Transcriptomic and Metabolomic Analysis Reveals the Impact of Autophagy Regulation on Purine Content in Mutton

**DOI:** 10.3390/foods14050718

**Published:** 2025-02-20

**Authors:** Xu Han, Yang Chen, Dandan Tan, Cuiyu Lai, Xuewen Han, Jinlin Chen, Yu Fu, Xuesong Shan, Huaizhi Jiang

**Affiliations:** College of Animal Science and Technology, Jilin Agricultural University, Changchun 130118, China; 15304283015@163.com (X.H.); chenyang7419@163.com (Y.C.); 13850139585@163.com (D.T.); 19843927673@163.com (C.L.); hanxuewen1123@163.com (X.H.); chenjinlin7314@163.com (J.C.); 15164460626@163.com (Y.F.)

**Keywords:** sheep, purine, autophagy, LAPTM5, XDH, HPRT

## Abstract

Background: Excessive purine intake increases the risk of hyperuricemia and gout. This study investigates the relationship between purine content in mutton and meat quality traits and explores the regulatory mechanism of purine metabolism through transcriptomic and metabolomic analyses. Methods: Thirty-six-month-old hybrid sheep (Australian White × Small-tail Han) were selected. Purine content and meat quality traits, including inosine monophosphate (IMP), intramuscular fat (IMF), shear force, pH, cooking loss, and meat color, were measured. Transcriptomic sequencing and metabolomic analysis were performed on muscle samples with high (3895.70 ± 107.03 mg/kg) and low (2751.72 ± 175.29 mg/kg) purine contents (*n* = 6 per group). Differentially expressed genes were validated by quantitative PCR and Western blot. In vivo autophagy regulation experiments were performed on mice using rapamycin (activator) and chloroquine (inhibitor). Results: No significant correlation was found between purine content and meat quality traits, suggesting that reducing purine content does not negatively affect mutton quality. An autophagy-related gene, LAPTM5 (lysosomal-associated protein transmembrane 5), was identified as a key gene negatively regulating purine content. In vivo autophagy regulation experiments demonstrated that autophagy affects purine metabolism by modulating key enzymes such as xanthine dehydrogenase (XDH) and hypoxanthine-guanine phosphoribosyltransferase 1 (HPRT). Conclusions: This study reveals the role of autophagy in regulating purine metabolism through the key enzymes XDH and HPRT, providing new insights for improving mutton quality in the future.

## 1. Introduction

Mutton is a significant component of the world’s major meats and holds a vital position in the culinary traditions of numerous countries and regions [1]. Its distinctive aroma and rich nutritional profile make it a favored food choice. However, the high purine content in mutton has led to increasing awareness and concern regarding food health among consumers [2]. According to the measurements in this study, the total purine content of low-purine lamb meat was 2751.72 ± 175.29 mg/kg, while the high-purine group had a content as high as 3895.70 ± 107.03 mg/kg. Purines, essential compounds for nucleic acid metabolism, are metabolized into uric acid in the body. Elevated levels of uric acid are closely linked to various health concerns, including gout and kidney disorders [3].

Currently, most research on purine contents in food focuses on reducing purine levels in mutton through physical methods to mitigate the incidence of hyperuricemia and gout. However, a more fundamental and meaningful approach is to directly produce low-purine mutton through interventions in animal production practices. Although feed has a limited impact on the purine content in mutton, the differences in purine concentrations across breeds indicate that breeding low-purine sheep is a viable solution. This requires a thorough understanding of the molecular genetic mechanisms that regulate purine metabolism in mutton, ensuring that meat quality remains unaffected while purine levels are reduced [4].

Although some studies have indicated a relationship between purine content and meat quality characteristics, the specific mechanisms remain unclear. Some studies suggest that purine content may indirectly affect the sensory characteristics of meat by influencing moisture retention and the formation of flavor compounds. However, there is limited research on the direct impact of purine content on meat quality. Therefore, systematically analyzing the relationship between purine levels and meat quality traits in mutton is particularly important [5]. Given that purine serves as a precursor to inosine monophosphate (IMP), a key flavor compound in mutton, we first demonstrated that a moderate reduction in purine content does not negatively affect meat quality through systematic analysis of the relationship between purine levels and meat quality indicators (including pH, shear force, cooking loss, and meat color). Building on this result, we further explored the specific mechanisms through which purine content affects meat quality in sheep.

Purines are essential molecules involved in a wide range of critical biological processes, including DNA and RNA synthesis, energy metabolism, and cellular signaling. As fundamental building blocks of life, purines play a pivotal role in regulating cellular activities and maintaining metabolic homeostasis. Given their centrality in cellular functions, the regulation of purine metabolism is highly complex and likely involves extensive gene expression and metabolic control mechanisms. To comprehensively unravel the regulatory network of purine metabolism, this study employed transcriptomic sequencing and metabolomic analysis to conduct a differential analysis of high- and low-purine mutton samples, aiming to identify key metabolic pathways and regulatory mechanisms.

LAPTM5 (lysosomal-associated protein transmembrane 5), an important lysosomal membrane protein involved in autophagy [6], was identified as a differentially expressed gene through the transcriptomic sequencing of high- and low-purine mutton samples. We hypothesized that LAPTM5 might regulate purine metabolism through autophagy, and notably, the relationship between LAPTM5 and purine metabolism has never been reported before. To test this hypothesis, we used experimental mice as a model organism for in vivo validation. The results not only confirmed that autophagy significantly affects muscle purine content but also revealed that the expression of two key enzymes involved in purine metabolism, xanthine dehydrogenase (XDH) and hypoxanthine-guanine phosphoribosyltransferase 1 (HPRT), are regulated by autophagy. This provides compelling evidence that autophagy may influence purine metabolism through these enzymes, ultimately affecting the purine content in muscle tissue.

By elucidating the molecular pathways through which autophagy regulates purine content, this study not only advances our understanding of purine metabolism in meat but also lays the groundwork for breeding low-purine sheep. Ultimately, this research has the potential to offer valuable insights for both human health and food science, particularly in the context of dietary purine management.

## 2. Materials and Methods

### 2.1. Correlation Between Purine Content and Taste Quality of Mutton

#### 2.1.1. Animals

Thirty hybrid ewes of small-tailed Han sheep and Australian White sheep, randomly selected for similar weights at six months of age, were raised at the Guofeng Sheep Breeding Farm in Changling County, Jilin Province, China, following the standards of the National Research Council (NRC) (2007). All sheep were fasted overnight before slaughter, and the *Longissimus dorsi* muscle from the left carcass of each sheep was collected. The animal protocol was approved by the Ethics Committee of Jilin Agricultural University.

#### 2.1.2. Quantification of Muscle Purine

Approximately 1 g of *Longissimus dorsi* muscle sample was taken (*n* = 30), and 2 mL of trifluoroacetic acid and 1 mL of formic acid were added. The mixture was thoroughly mixed and hydrolyzed at 80 °C for 60 min. After hydrolysis, it was cooled to room temperature and diluted to 25 mL. A 2 mL aliquot of the sample was taken, and potassium dihydrogen phosphate was added to adjust the pH to 4; then, it was diluted to 4 mL, filtered, and prepared for analysis using a Thermo Fisher U3000 HPLC (Waltham, MA, USA). The chromatographic conditions included a Thermo Fisher Hypersil GOLD™ C18 column (250 mm × 4.6 mm, 5 μm) with a mobile phase of water (0.05 mol/L, pH = 4.00) at a wavelength of 254 nm.

Based on the results of purine determination, we selected six sheep samples with the highest purine content (H) and six sheep samples with the lowest purine content (L), respectively, at a ratio of 20%. We conducted a differential analysis of purine content and subsequent transcriptomic and metabolomic analyses.

#### 2.1.3. Determination of Meat Quality Indicators

*Longissimus dorsi* muscle tissue samples (*n* = 30) were collected for meat quality determination. The indicators measured included pH value, shear force, cooking loss, meat color, inosine monophosphate (IMP), and intramuscular fat (IMF) content. The determination of each meat quality index followed the guidelines outlined in the “Operation Manual for the Third National Census of Livestock and Poultry Genetic Resources (First Edition)” [7].

### 2.2. Transcriptome and Metabolome Sequencing

#### 2.2.1. Transcriptome Sequencing

Total RNA was extracted from muscle tissue using TRIzol reagent (Pudong New Area, Shanghai, China), following the manufacturer’s instructions. RNA purity and concentration were assessed using a NanoDrop 2000 spectrophotometer (Thermo, USA) and an Agilent 2100 bioanalyzer (Agilent Technologies, Santa Clara, CA, USA). The RNA library was constructed and sequenced on the Illumina PE150 platform. Raw data were subjected to quality filtering to obtain high-quality reads, which were then aligned to the sheep genome data obtained from the NCBI database. The reads were assembled and spliced using Trinity 2.8.4 software, and gene expression levels were quantified using the TPM (Transcripts Per Million) method.

#### 2.2.2. Metabolome Sequencing

Further, 50 mg *Longissimus dorsi* muscle tissue was placed in a 2 mL centrifuge tube, and 400 μL of extraction solution (methanol = 4:1) was added to extract metabolites. After homogenization, low-temperature ultrasonic extraction, standing, and centrifugation, the supernatant was transferred to a sample injection vial for analysis. All samples were analyzed by LC-MS, with six biological replicates per group.

#### 2.2.3. Transcriptomic and Metabolomic Data Analysis

Differential expression analysis of genes between groups was performed using DESeq2, and the fold change (FC) of each gene was calculated. Differentially expressed genes (DEGs) were selected based on the criteria of |log_2_FC| ≥ 1. KEGG enrichment analysis was conducted to identify enriched pathways associated with DEGs. Principal component analysis (PCA) was performed to evaluate the differences between samples. Metabolic pathway annotation was conducted using the KEGG database, and the pathway enrichment analysis of both differentially expressed genes and metabolites was performed using the same database.

### 2.3. Immunoblotting

Muscle protein was extracted using a whole protein extraction kit, and the protein concentration was determined using the BCA assay. SDS-PAGE gel preparation was performed according to the standard protocol, and protein samples were loaded for electrophoresis. Semi-dry transfer was carried out at constant current for 34 min. The membrane was incubated overnight at 4 °C with the primary antibody (1:1000 dilution). Following incubation with the secondary antibody the next day, protein bands were visualized using a gel imaging system. Grayscale values were quantified using ImageJ software (v1.8.0, National Institute, Bethesda, MD, USA).

### 2.4. Fluorescence Quantitative PCR

Specific primers were designed using Primer Premier 5.0 software based on the sheep gene sequences available in the NCBI database. Sheep muscle tissue was collected, ground in liquid nitrogen, and total RNA was extracted using the Trizol reagent. cDNA synthesis was carried out following the instructions of the reverse transcription kit, and quantitative PCR was performed (Appendix A). The relative gene expression was calculated using the 2^−ΔΔCt^ method.

### 2.5. GSEA

Gene Set Enrichment Analysis (GSEA) was performed to assess gene functional enrichment between the two groups. GSEA identifies gene sets with coordinated differential expressions from the overall gene expression matrix, thus accounting for genes with small differential changes. The following parameters were used: “C2.KEGG pathway gene set” was selected as the background, with 1000 permutations as the default number for the significance test. For smaller sample sizes, the gene set permutation test was selected, and gene names were used for gene type. Due to the sample size being greater than three, the ranking method for genes was set to Signal2Noise, with other parameters set to their default values.

### 2.6. Autophagy Regulation in Mice

Mice and sheep exhibit numerous similarities in their metabolic pathways, encompassing glucose metabolism, fat metabolism, and protein metabolism. Autophagy plays a crucial role in modulating purine metabolic enzymes, a process that is prevalent in both mice and sheep. Six-week-old Kunming mice were randomly divided into three groups: the control group, chloroquine-induced autophagy inhibition group, and rapamycin-induced autophagy activation group, with 10 mice in each group. The mice were intraperitoneally injected daily with 0.25 mL of physiological saline, chloroquine (60 mg/kg) [8], or rapamycin (1 mg/kg) [9] for 21 consecutive days. At the end of the 21-day period, the mice were euthanized, and the *Longissimus dorsi* muscle was collected. Purine content measurement and transcriptome sequencing were performed according to the methods described in Section 2.1.1 and Section 2.2.1.

### 2.7. Data Analysis

Data were organized using Microsoft Excel, and the results are presented as “mean ± standard error”. Statistical analyses were performed using SPSS 26.0 software. Prior to selecting the statistical methods, the Shapiro–Wilk test was performed to assess the distribution of the data. Independent sample *t*-tests and one-way ANOVA were used to compare group differences, with a significance level set at *p* < 0.05 for significant differences, *p* < 0.01 for highly significant differences, and *p* > 0.05 for no significant difference. Graphs were generated using GraphPad Prism 8.0.2 software.

## 3. Results and Analysis

### 3.1. Correlation Between Purine Content and Meat Quality

**Figure 1:**
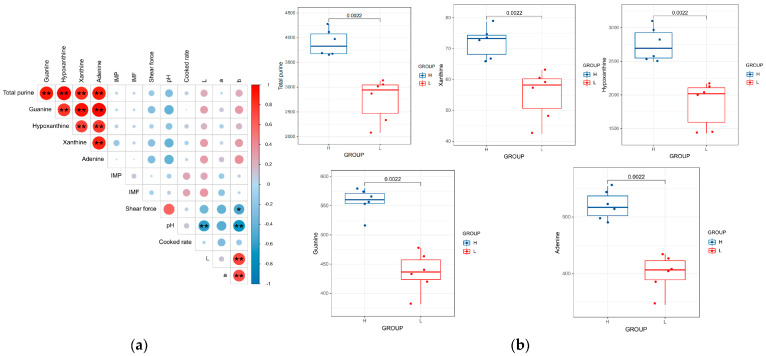
Relationship between purine content and meat quality traits. (**a**) Heatmap showing the correlation between purine content and meat quality traits. (**b**) Comparison of the four purine bases and total purine content (mg/kg) in the *Longissimus dorsi* muscle between the high-purine (H) and low-purine (L) groups, along with the inter-group differences. * indicates *p* < 0.05, ** indicates *p* < 0.01.

Purine content and meat quality traits (IMP, IMF, shear force, pH, cooking loss, and meat color) were measured in the *Longissimus dorsi* muscle of 30 lambs, and Pearson correlation coefficients were calculated (Figure 1a). The results showed no significant correlation between purine content and meat quality traits (*p* > 0.05), indicating that the contents of different purine bases in lamb meat do not negatively affect meat quality traits. Although the differences were not statistically significant, a moderate reduction in purine content showed a trend towards improved tenderness (lower shear force) and increased flavor compounds (IMP), suggesting a potential improvement in lamb meat quality. This finding supports the feasibility and significance of lowering the purine content in lamb.

The levels of the four purine bases and their correlation with total purine content were highly significant (*p* < 0.001). Therefore, in subsequent analyses, total purine content was used as the primary measure. Based on the total purine content, the experimental animals were divided into high-purine (H, *n* = 6) and low-purine (L, *n* = 6) groups. The total purine content in the high-purine group was 3895.70 ± 107.03 mg/kg, while the total purine content in the low-purine group was 2751.72 ± 175.29 mg/kg. The highest content was found in hypoxanthine, with concentrations of 2746.48 ± 100.93 mg/kg in the high-purine group and 1869.71 ± 136.98 mg/kg in the low-purine group. Significant differences in the levels of the four purine bases and total purine content were observed between the two groups (*p* < 0.01) (Figure 1b), confirming the validity of the experimental grouping and justifying the use of these groups for subsequent transcriptomic and metabolomic analyses.

### 3.2. Transcriptomic Analysis

**Figure 2:**
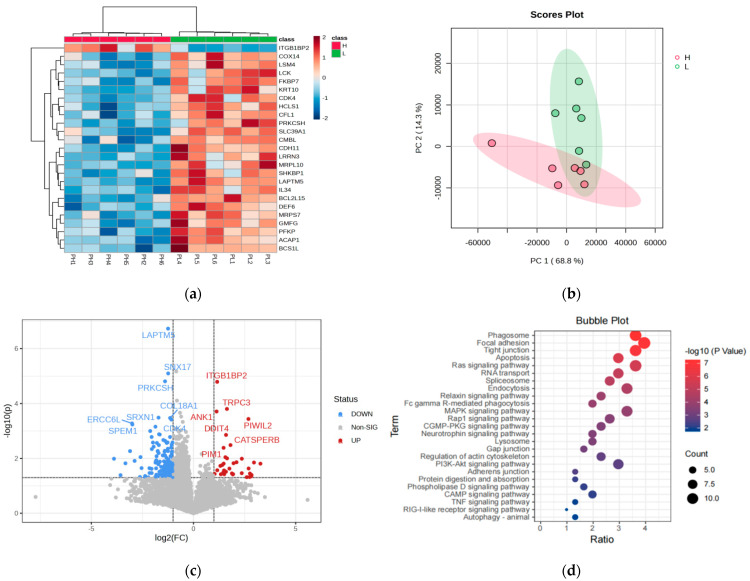
Transcriptomic analysis of high-purine group (H) and low-purine group (L). (**a**) Heatmap of gene expression and sample clustering, (**b**) principal component analysis (PCA) plot, (**c**) volcano plot of differentially expressed genes, (**d**) KEGG pathway enrichment analysis of differentially expressed genes.

The clustering heatmap of differentially expressed genes (DEGs) between the high-purine (H) and low-purine (L) groups reveals the distinct clustering of the two sample groups (Figure 2a), suggesting that the clustered genes may share similar functional annotations or belong to the same metabolic pathway. Moreover, there were clear expression differences between the groups. The principal component analysis (PCA) results (Figure 2b) show that the samples from the high-purine and low-purine groups were clearly separated, with samples from each group clustering near the center, indicating distinct differences between the two groups and the good reproducibility of the analysis.

Compared to the low-purine group (L), a total of 209 differentially expressed genes (DEGs) were identified in the high-purine group (H), with 25 genes upregulated (11.96% of the total DEGs) and 184 genes downregulated (88.04% of the total DEGs) (Figure 2c).

KEGG enrichment analysis of the 302 differentially expressed genes (DEGs) identified 81 enriched metabolic pathways, of which 26 pathways were significantly enriched (*p* < 0.05). The top 25 significantly enriched KEGG pathways are shown in Figure 2d. Notably, pathways such as the autophagy signaling pathway, apoptosis, RAS signaling pathway, focal adhesion kinase signaling pathway, and MAPK signaling pathway may be related to purine content. Among them, the autophagy pathway, represented by phagosomes, showed the highest significance. Autophagy and apoptosis are mutually regulated, and they play a crucial role in determining cell survival or death under cellular stress. The RAS pathway influences autophagic activity by regulating cell proliferation and survival, while the MAPK pathway modulates the expression of autophagy-related genes.

### 3.3. Metabolomic Analysis

**Figure 3:**
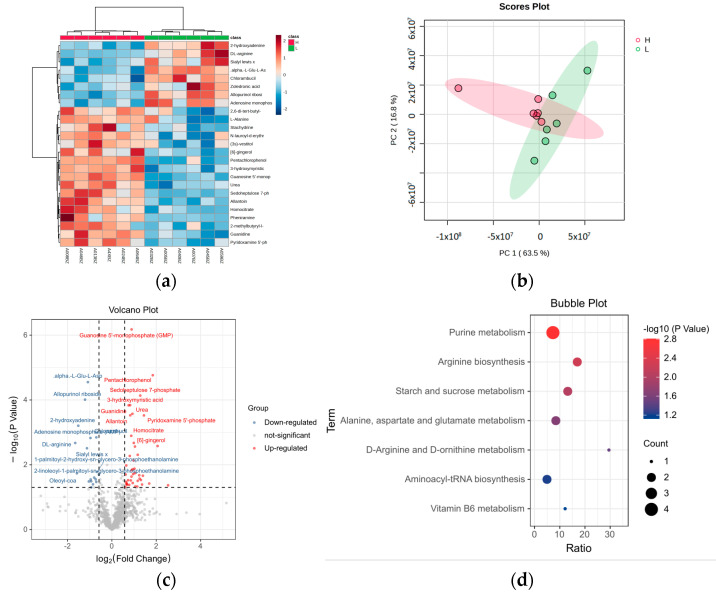
Metabolomic analysis of high-purine group (H) and low-purine group (L). (**a**) Heatmap of metabolite expression and sample clustering, (**b**) principal component analysis (PCA) plot, (**c**) volcano plot of differential metabolites, (**d**) KEGG pathway enrichment analysis of differential metabolite.

Clustering analysis of differential metabolites between the high-purine (H) and low-purine (L) groups revealed the distinct separation of the two sample groups (Figure 3a), suggesting that the clustered metabolites may share similar functional annotations or belong to the same metabolic pathway, with clear metabolic differences between the groups. The principal component analysis (PCA) results (Figure 3b) show that the samples from the high-purine and low-purine groups were clearly separated, indicating metabolic differences between the two groups, with good reproducibility of the analysis.

Compared to the low-purine group (L), a total of 59 differential metabolites (DMs) were identified in the high-purine group (H), with 42 metabolites upregulated and 17 metabolites downregulated (Figure 3c). KEGG enrichment analysis of the 59 differential metabolites revealed the top 7 significantly enriched metabolic pathways (*p* < 0.05), as shown in (Figure 3d). The KEGG pathway analysis indicated that the differential metabolites were primarily enriched in purine metabolism, arginine biosynthesis, starch and sucrose metabolism, and alanine, aspartate, and glutamate metabolism, among others.

### 3.4. Differential Expression of Key Purine Metabolism Genes Between High-Purine Group and Low-Purine Groups

**Figure 4:**
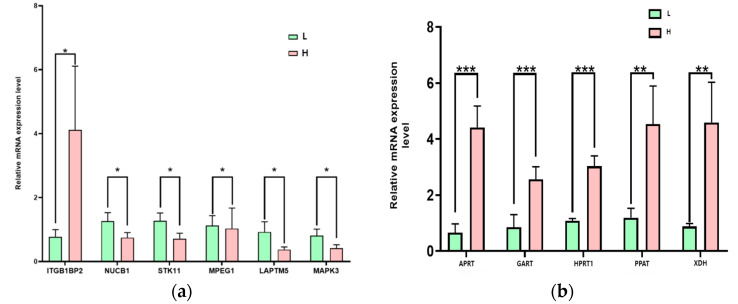

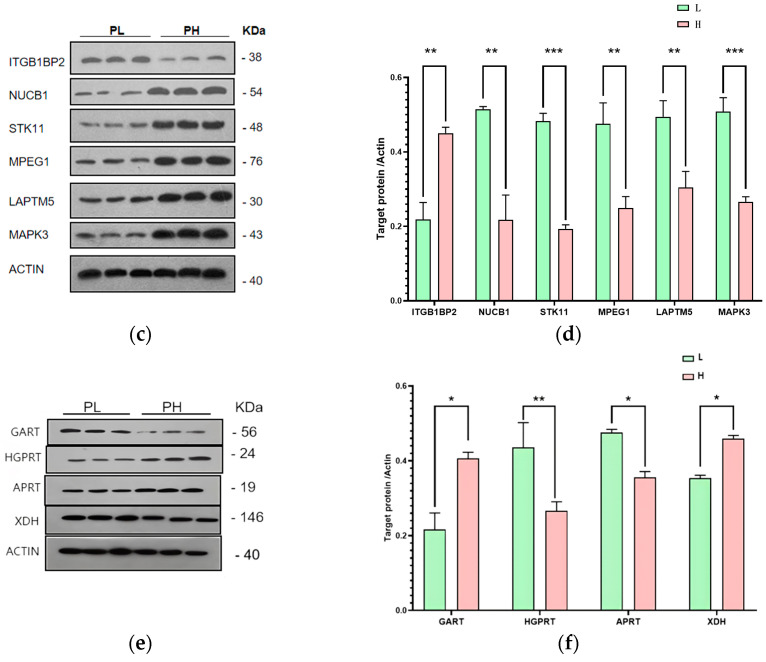
Differential expressions of genes and key purine metabolism enzymes in high-purine group (H) and low-purine group (L). (**a**) Quantitative PCR of differentially expressed genes (**b**) and (**c**) Western blot analysis of differentially expressed genes; (**d**) quantitative PCR of key purine metabolism enzymes; (**e**,**f**) Western blot analysis of key purine metabolism enzymes. * indicates *p* < 0.05, ** indicates *p* < 0.01, and *** indicates *p* < 0.001.

Six differentially expressed genes were randomly selected for validation using quantitative PCR and Western blot (WB). The results (Figure 4a,b) show that the transcription levels and protein expression of Neuropeptide precursor protein 1 (NUCB1), Serine/threonine kinase 11 (STK11), Macrophage-expressed gene 1 (MPEG1), Lysosomal-associated protein transmembrane 5 (LAPTM5), and Mitogen-activated protein kinase 3 (MAPK3) were significantly higher in the low-purine group compared to the high-purine group (*p* < 0.05). In contrast, the transcription levels and protein expression of Integrin beta-1 binding protein 2 (ITGB1BP2) were significantly higher in the high-purine group compared to the low-purine group (*p* < 0.05). These results were consistent with the transcriptomic analysis, confirming the reliability of the sequencing data.

Additionally, to explore the regulatory mechanisms of the muscle purine content, the transcriptional and protein expression levels of three key enzymes involved in purine metabolism—from de novo synthesis, salvage pathways, and purine degradation—were analyzed [10]. The results show that the expression levels of Phosphoribosylglycinamide transformylase (GART), Adenine phosphoribosyltransferase (APRT), and Xanthine dehydrogenase (XDH) in the low-purine group were significantly lower than those in the high-purine group (*p* < 0.05). Moreover, the transcriptional and protein levels of Hypoxanthine-guanine phosphoribosyltransferase 1 (HPRT) in the low-purine group were significantly lower than those in the high-purine group (*p* < 0.01).

### 3.5. Target Gene Identification and GSEA

**Figure 5:**
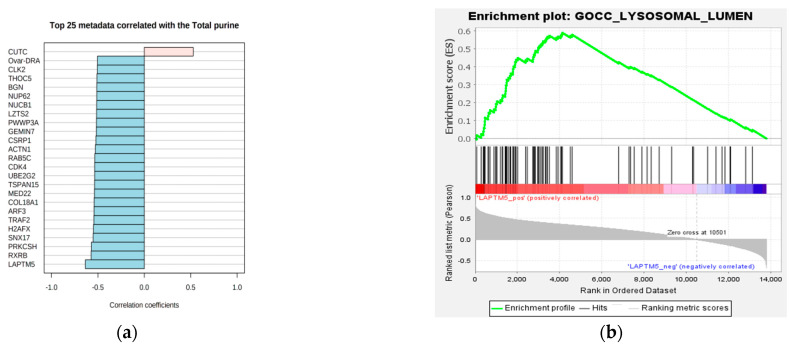
Correlation between gene expression and purine content. (**a**) Correlation analysis of gene expression and purine content (TOP 25), (**b**) GSEA enrichment of the LAPTM5 gene in the lysosome pathway.

To more effectively identify the target gene, we not only screened differentially expressed genes using transcriptomic sequencing but also calculated gene expression levels and their correlation with total purine content (Figure 5a). We found that the LAPTM5 gene exhibited the most significant expression difference between the high-purine and low-purine groups (FC of H/L = 0.45) and showed the highest correlation with purine content (R = −0.6353, *p* = 0.000162). Therefore, LAPTM5 was selected as the target gene for subsequent experiments. LAPTM5 (Lysosomal-associated transmembrane protein 5) is a lysosomal multi-transmembrane protein primarily located on the lysosomal membrane [11]. To explore the function of LAPTM5, we conducted GSEA using transcriptomic data, which revealed 40 enriched metabolic pathways. Notably, both the GSEA and transcriptomic KEGG enrichment analyses identified the autophagy pathway (Figure 5b), suggesting that LAPTM5 may influence purine content by modulating autophagy.

### 3.6. Autophagy Differences Between High-Purine and Low-Purine Groups

**Figure 6:**
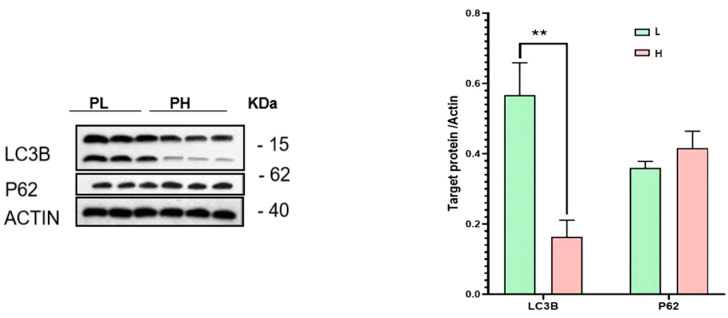
Western blot analysis of autophagy markers in high-purine (H) and low-purine groups (L). ** indicates *p* < 0.01.

Given that LAPTM5 is associated with the lysosomal autophagy pathway, we measured the expression levels of autophagy markers P62 and LC3B in the high-purine (H) and low-purine (L) groups (Figure 6) [12]. The results show that in the low-purine group, LC3B II levels were decreased, indicating a reduction in autophagosome formation, while P62 levels were increased, suggesting a blockade in the degradation of autophagosomes. These findings imply that autophagosome formation in the low-purine group may be inhibited, preventing autophagosomes from entering the lysosomal degradation pathway [13]. Overall, autophagy flux was suppressed in the low-purine group. This, combined with the observation that LAPTM5 is involved in autophagy and negatively correlated with purine content, further supports the hypothesis that LAPTM5 may lower muscle purine content by promoting autophagy [14].

### 3.7. Effects of Autophagy Activators and Inhibitors on Purine Content in Mouse Muscle

To further validate the hypothesis that autophagy levels influence purine content, we conducted an autophagy modulation in mice and measured the purine levels in their muscle tissues. The results indicate that the purine levels of xanthine and guanine in the muscle of mice treated with the autophagy activator rapamycin and the autophagy inhibitor chloroquine were both lower than those in the control group. Specifically, the xanthine content in the rapamycin group decreased significantly by 34.84% compared to the control group (*p* < 0.01). Although not reaching statistical significance (*p* > 0.05), the xanthine content in the chloroquine group was reduced by 17.07% compared to the control group.

The guanine content in the muscle of both the rapamycin and chloroquine groups did not show significant differences compared to the control group (*p* > 0.05). Additionally, the levels of adenine, hypoxanthine, and total purine content in the muscle of the rapamycin and chloroquine groups were higher than those in the control group. Notably, the hypoxanthine content in the chloroquine group was significantly elevated by 42.19% compared to the control group (*p* < 0.05), while the xanthine levels in the rapamycin group did not show significant differences from the control group (Table 1).

### 3.8. Autophagy Regulation in Mouse Transcriptome Sequencing and Western Blotting

**Figure 7:**
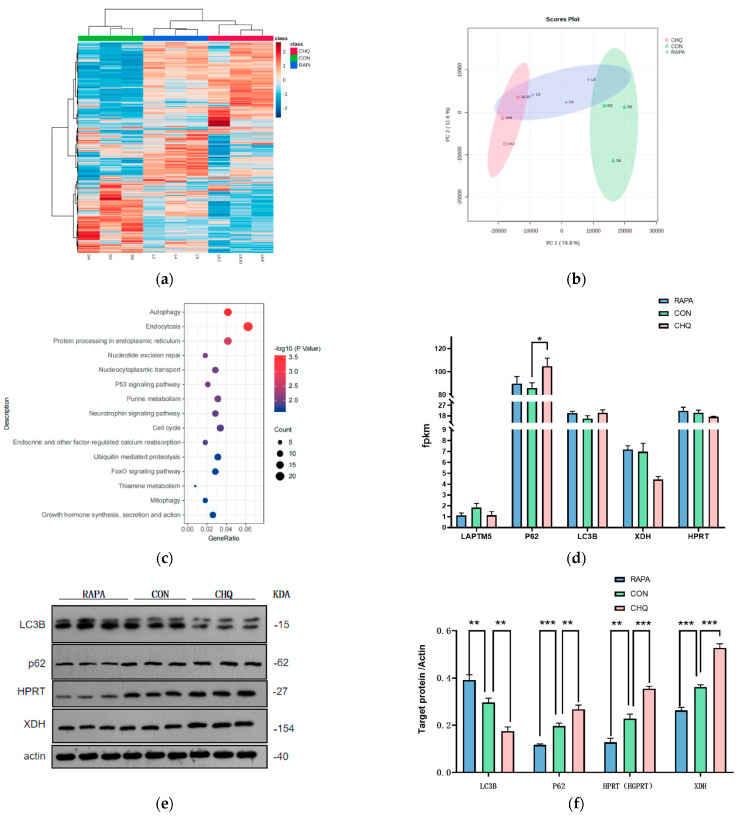
Transcriptome sequencing and western blotting of the longest back muscle in mice under different treatments. (**a**) Heatmap of gene expression and sample clustering, (**b**) PCA plot, (**c**) KEGG enrichment analysis of differentially expressed genes, (**d**) transcriptome gene expression levels, (**e**) Western blot of autophagy marker proteins (**f**) Western blot of purine metabolizing enzymes. (RAPA: Rapamycin, CON: contrast, CHQ: chloroquine). * indicates *p* < 0.05, ** indicates *p* < 0.01, and *** indicates *p* < 0.001.

Transcriptome sequencing was performed on the longest back muscle of mice from the autophagy activation group, autophagy inhibition group, and control group. Gene expression clustering analysis and PCA results indicate significant differences in gene expression patterns among the three treatment groups (Figure 7a,b). The KEGG enrichment analysis of differentially expressed genes highlighted pathways related to autophagy, endocytosis, protein processing in the endoplasmic reticulum, nucleotide excision repair, nucleocytoplasmic transport, the P53 signaling pathway, purine metabolism, neurotrophic factor signaling pathways, cell cycle regulation, endocrine factors, and calcium reabsorption (Figure 7c) [15]. These enrichment results confirm the effectiveness of autophagy modulation and demonstrate that autophagy status indeed regulates purine metabolism [16]. The Western blot results further corroborate these findings (Figure 7d,e): in the rapamycin treatment group, LC3 expression was elevated while P62 expression decreased, indicating enhanced autophagy; conversely, in the chloroquine treatment group, LC3B levels decreased and P62 levels increased, indicating inhibited autophagy. Notably, in the transcriptome differential analysis (Figure 7c), P62 protein, an autophagy substrate, was significantly elevated in the autophagy inhibition group (*p* < 0.05), while the expression differences in the genes LAPTM5, LC3B, XDH, and HPRT were not significant (*p* > 0.05). The lack of significant difference in LAPTM5 gene expression suggests that autophagy does not influence LAPTM5 expression, supporting our hypothesis that LAPTM5 affects autophagy rather than vice versa. Furthermore, the insignificant differences in XDH and HPRT expression indicate that autophagy regulation of these proteins occurs not at the transcriptional level but rather through lysosomal degradation pathways [17]. The Western blot results confirm this finding, showing significant differences in the expression of the LC3B, XDH, and HPRT proteins among the groups (*p* < 0.01).

## 4. Discussions

This study explored the relationship between purine content and meat quality traits in sheep meat. It was found that there is no significant correlation between purine content and meat quality characteristics. This finding suggests that reducing purine levels in sheep meat may not adversely affect meat quality and could potentially enhance certain characteristics.

To elucidate the molecular basis of purine content regulation in muscle, we identified differentially expressed genes and metabolites in muscle from various purine content groups through transcriptomic and metabolomic analyses. The results indicate that the transcriptome was primarily enriched in autophagy pathways, while the metabolome was mainly enriched in purine metabolism. Notably, LAPTM5 emerged as a key functional gene, with its expression negatively correlated with purine content (R = −0.6353, *p* = 0.000162). Further Gene Set Enrichment Analysis (GSEA) and KEGG enrichment analyses highlighted the significant role of LAPTM5 in the autophagy process. The Western blot results showed that higher LAPTM5 expression was associated with enhanced autophagy. Consistent with our conclusions, studies by other researchers also suggest that LAPTM5 plays an important role in the autophagy process. A study by Yang, K. (2024) found that LAPTM5 can drive lenvatinib resistance by promoting autolysosome formation, thus enhancing intrinsic macroautophagy/autophagic flux [6]. Another study by Jiang L et al. (2023) indicated that LAPTM5 promotes the transport and degradation of CDC42 to the lysosome [18]. Although no studies have reported on the relationship between LAPTM5 and purine metabolism, based on LAPTM5’s role in regulating autophagy, we speculate that LAPTM5 may influence purine metabolism through autophagy regulation.

To validate this hypothesis, an animal experiment was conducted using autophagy regulation in mice. The mice were treated with rapamycin and chloroquine to modulate autophagy levels. In the rapamycin-treated group, the levels of xanthine significantly decreased, while hypoxanthine and adenine levels increased, and guanine levels decreased. In the chloroquine-treated group, xanthine levels decreased, while hypoxanthine levels significantly increased, and adenine levels increased, with guanine levels decreasing. The overall trends were similar, with the primary differences observed in hypoxanthine and xanthine levels. Transcriptome analysis of the rapamycin-treated, chloroquine-treated, and control groups shows that although the autophagy levels were altered by the drugs, there was no difference in LAPTM5 gene expression. This demonstrates that changes in autophagy levels did not affect LAPTM5 expression, confirming that LAPTM5 influences purine content via autophagy.

The oxidation and subsequent metabolism of purine nucleotides into uric acid is a complex process, summarized by two main pathways: (1) Guanosine triphosphate (GTP) → Guanosine monophosphate (GMP) → Guanine → Xanthine → Uric acid; (2) Adenosine triphosphate (ATP) → Adenosine monophosphate (AMP) → Inosine monophosphate (IMP) → Hypoxanthine → Xanthine → Uric acid, with the second pathway being predominant [19]. Western blotting and q-PCR analysis across the three treatment groups revealed that HPRT and XDH protein levels significantly decreased in the rapamycin treatment group, while they increased in the chloroquine treatment group. HPRT (hypoxanthine/guanine phosphoribosyl transferase) primarily participates in the purine salvage pathway, converting hypoxanthine to IMP and guanine to GMP. XDH (xanthine dehydrogenase) is a key enzyme in purine metabolism, involved in the oxidation of hypoxanthine and xanthine, ultimately generating uric acid. Our findings indicate that the autophagy activator rapamycin significantly reduced xanthine levels in muscle, suggesting that autophagy affects the conversion of hypoxanthine to xanthine and the purine salvage pathway. Western blotting results confirmed that the expression levels of key purine metabolic enzymes XDH and HPRT were significantly lower in the autophagy activation group (*p* < 0.01), demonstrating that rapamycin-induced autophagy inhibits the purine salvage pathway and the conversion of hypoxanthine to xanthine. The chloroquine treatment group, which inhibits autophagy, promoted the purine salvage pathway, facilitating the conversion of hypoxanthine to xanthine. The inhibition of autophagy led to the accumulation of hypoxanthine, increasing its conversion to hypoxanthine nucleotides [20]. Yang, K. et al. (2024) found that HPRT targets purine metabolism and influences autophagic flux through NR4A1 [21]. Liu, D. et al. (2024) showed that HPRT is degraded through the lysosomal pathway [22]. These studies provide supporting evidence for our hypothesis that autophagy affects the expression of XDH and HPRT, thereby influencing purine metabolism.

Based on the findings outlined in the preceding research, we propose that LAPTM5 plays a pivotal role in facilitating autophagy. This process, in turn, influences the degradation and recycling of intracellular substances, thereby supplying essential substrates and regulatory factors that contribute to purine metabolism. Furthermore, LAPTM5 may also regulate purine metabolism by modulating the expression of genes or proteins directly involved in this metabolic pathway. Specifically, autophagy predominantly impacts purine metabolic enzymes, with a notable effect on those involved in the purine salvage pathway and the interconversion between hypoxanthine and xanthine.

The implications of these findings are profound, especially regarding the prevention and treatment of gout. Xanthine dehydrogenase (XDH) and hypoxanthine-guanine phosphoribosyltransferase (HPRT) emerge as two promising therapeutic targets for gout. Hypoxanthine is the most prevalent purine form in muscle tissue, while elevated levels of xanthine serve as a major trigger for gout [23]. Xanthine is subsequently converted into uric acid, and its excessive accumulation is a primary cause of gout and hyperuricemia. Currently, allopurinol, a drug that mimics hypoxanthine-like compounds, is commonly used to manage gout [24]. Allopurinol inhibits xanthine oxidase by competitively binding to it, thereby reducing uric acid production. Additionally, allopurinol interacts with PRPP (5′-phosphoribosyl-1-pyrophosphate) to form allopurinol nucleotides, which not only depletes PRPP but also provide negative feedback to suppress enzymes involved in the de novo synthesis of purine nucleotides. This action results in a reduction in purine nucleotide synthesis. Our findings are consistent with the mechanism of action of allopurinol, as both mechanisms reduce the conversion of hypoxanthine to xanthine and exert negative feedback on purine nucleotide synthesis, providing a novel approach for gout treatment.

Moreover, the insights derived from this study hold substantial value for the livestock and meat industries. Our findings directly support the development of low-purine sheep meat through selective breeding. This could potentially reduce the purine content in meat products, which may offer benefits for human health, particularly in preventing purine-related disorders such as gout.

Our study has several limitations. First, the relatively small sample size may limit the generalizability of our findings. Although we performed inter-group difference tests, we recognize that a larger sample size would yield more robust and convincing results. Additionally, while transcriptomic analysis identified LAPTM5 as the gene most significantly correlated with muscle purine content, and we hypothesized that LAPTM5 reduces purine levels by promoting autophagy, we did not directly demonstrate how LAPTM5 regulates purine metabolism. Although we validated in vivo that autophagy influences purine content, future studies should utilize LAPTM5 overexpression or silencing models at both cellular and animal levels to further elucidate the role and regulatory network of LAPTM5 in purine metabolism.

## 5. Conclusions

This study explored the relationship between meat quality traits and purine content, finding that appropriately reducing purine levels in meat does not compromise overall quality, including taste, flavor, and tenderness. By breeding low-purine sheep breeds, it is possible to meet the needs of specific consumer groups who require limited purine intake for health reasons. Furthermore, this study confirmed that autophagy can influence purine content by affecting the key metabolic enzymes XDH and HPRT, particularly the levels of hypoxanthine and xanthine, with LAPTM5 potentially playing a crucial role in this process.

## Figures and Tables

**Table 1:** Purine content in mouse muscle among different groups (Mean ± SD).

	Rapamycin	Control	Chloroquine
xanthine	20.51 ± 6.29 **	31.48 ± 4.74	26.10 ± 5.6
adenine	142.31 ± 21.52	139.39 ± 26.48	155.99 ± 18.82
hypoxanthine	916.81 ± 184.88	705.12 ± 234.65	1002.68 ± 113.77 *
guanine	152.92 ± 25.98	184.79 ± 43.84	167.98 ± 28.92
total purines	1232.56 ± 230.22	1060.78 ± 295.46	1352.74 ± 136.45 *

* *p* < 0.05 ** *p* < 0.01.

## Data Availability

The original contributions presented in this study are included in the article. Further inquiries can be directed to the corresponding authors.

## Abbreviations

IMF	Inosine monophosphate
IMP	Intramuscular fat
PCA	Principal component analysis
GSEA	Gene set enrichment analysis
DMs	Differential metabolites
DGES	Differentially expressed genes
WB	Western Blot
ITGB1BP2	Integrin Subunit Beta 1 Binding Protein 2
NUCB1	Nucleobindin 1
STK11	Serine/threonine kinase 11
MPEG1	Macrophage expressed gene 1
LAPTM5	Lysosomal-associated protein transmembrane 5
MAPK3	Mitogen-activated protein kinase
PPAT	Phosphoribosyl pyrophosphate amidotransferase
GART	Phosphoribosyl glycinamide formyltransferase
HPRT	Hypoxanthine-guanine phosphoribosyltransferase
APRT	Adenine phosphoribosyltransferase
XDH	Xanthine dehydrogenase
RAPA	Rapamycin
CON	Contrast
CHQ	Chloroquine

## Appendix A. Fluorescence Quantitative PCR Primers

ITGB1BP2-F TGTCTCTACTCTGCCATAA
ITGB1BP2-R GGTAACAGGAATCAGGAAG
NUCB1-F AGACCTTCTTCATACTGC
NUCB1-R GTCATCGTCCTCATTCTT
STK11-F CAACGTGAAGAAGGAGAT
STK11-R TACAGCACATCCACTAAC
MPEG1-F CTCCAACTCCATCAACAT
MPEG1-R CCTGAAGGGTCTTTATCC
LAPTM5-F CTCATAACCAGTTCATCAAG2
LAPTM5-R CATCAGTCTGTAGCATCT
MAPK3-F CAGGATCTGATGGAGACA
MAPK3-R TGGTACAGGAAGTAGCATA
PPAT-F CTTCACCACCAATTAGAT
PPAT-R TTCAGTGTTATTCCTTCTT
GART-F AGAATCTACAGCCACTCA
GART-R AGTAATCCTCCACCAGTAA
HPRT-F ATCCATTCCTATGACTGTA
HPRT-R CGACAATCAAGACATTCT
APRT-F CTCGTATGCGTTGGAATA
APRT-R TCTACAACCACCACCTTC
XDH-F ATCTTCCTGCCATTATCAC
XDH-R ACCACATTATCTGCTTCTG

## References

[1] Ding W., Lu Y., Xu B., Chen P., Li A., Jian F., Yu G., Huang S. (2024). Meat of Sheep: Insights into Mutton Evaluation, Nutritive Value, Influential Factors, and Interventions. Agriculture.

[2] Zhao B., Sun B., Wang S., Zhang Y., Zang M., Le W., Wang H., Wu Q. (2021). Effect of different cooking water on flavor characteristics of mutton soup. Food Sci. Nutr..

[3] Ramos G.K., Goldfarb D.S. (2022). Update on uric acid and the kidney. Curr. Rheumatol. Rep..

[4] Huang C., Zheng M., Huang Y., Cai L., Zou X., Yao T., Xie X., Yang B., Xiao S., Ma J. (2024). Unraveling genetic underpinnings of purine content in pork. J. Integr. Agric..

[5] Zheng M., Huang Y., Ji J., Xiao S., Ma J., Huang L. (2018). Effects of breeds, tissues and genders on purine contents in pork and the relationships between purine content and other meat quality traits. Meat Sci..

[6] Pan J., Zhang M., Dong L., Ji S., Zhang J., Zhang S., Lin Y., Wang X., Ding Z., Qiu S. (2022). Genome-Scale CRISPR screen identifies LAPTM5 driving lenvatinib resistance in hepatocellular carcinoma. Autophagy.

[7] Wang X. (2021). Operation Manual for the Third National Census of Livestock and Poultry Genetic Resources (First Edition). China Anim. Husb..

[8] Doeppner T.R., Coman C., Burdusel D., Ancuta D.L., Brockmeier U., Pirici D.N., Yaoyun K., Hermann D.M., Popa-Wagner A. (2022). Long-term treatment with chloroquine increases lifespan in middle-aged male mice possibly via autophagy modulation, proteasome inhibition and glycogen metabolism. Aging.

[9] Luo D., Ren H., Li T., Lian K., Lin D. (2016). Rapamycin reduces severity of senile osteoporosis by activating osteocyte autophagy. Osteoporos. Int..

[10] Tran D.H., Kim D., Kesavan R., Brown H., Dey T., Soflaee M.H., Vu H.S., Tasdogan A., Guo J., Bezwada D. (2024). De novo and salvage purine synthesis pathways across tissues and tumors. Cell.

[11] Zhang M.M., Liang M.J., Zhang D.M., Cai J.N., Yang Q.J., Zhang J.P., Li Y.L. (2024). The function and mechanism of LAPTM5 in diseases. Biomed. Pharmacother..

[12] Hwang H.J., Ha H., Lee B.S., Kim B.H., Song H.K., Kim Y.K. (2022). LC3B is an RNA-binding protein to trigger rapid mRNA degradation during autophagy. Nat. Commun..

[13] Mahapatra K.K., Mishra S.R., Behera B.P., Patil S., Gewirtz D.A., Bhutia S.K. (2021). The lysosome as an imperative regulator of autophagy and cell death. Cell. Mol. Life Sci..

[14] Sun P.P., Liao S.X., Sang P., Liu M.M., Yang J.B. (2024). Lysosomal transmembrane protein 5: Impact on immune cell function and implications for immune-related deficiencies. Heliyon.

[15] Snytnikova O., Tsentalovich Y., Sagdeev R., Kolosova N., Kozhevnikova O. (2022). Quantitative Metabolomic Analysis of Changes in the rat blood serum during autophagy modulation: A focus on accelerated senescence. Int. J. Mol. Sci..

[16] Furuhashi M. (2020). New insights into purine metabolism in metabolic diseases: Role of xanthine oxidoreductase activity. Am. J. Physiol. Endocrinol. Metab..

[17] Tanaka T., Warner B.M., Michael D.G., Nakamura H., Odani T., Yin H., Atsumi T., Noguchi M., Chiorini J.A. (2022). LAMP3 inhibits autophagy and contributes to cell death by lysosomal membrane permeabilization. Autophagy.

[18] Jiang L., Zhao J., Yang Q., Li M., Liu H., Xiao X., Tian S., Hu S., Liu Z., Yang P. (2023). Lysosomal-associated protein transmembrane 5 ameliorates non-alcoholic steatohepatitis by promoting the degradation of CDC42 in mice. Nat. Commun..

[19] Burnstock G. (2020). Introduction to purinergic signaling. Purinergic Signaling: Methods and Protocols.

[20] Yang K.J., Park H., Chang Y.K., Park C.W., Kim S.Y., Hong Y.A. (2024). Xanthine oxidoreductase inhibition ameliorates high glucose-induced glomerular endothelial injury by activating AMPK through the purine salvage pathway. Sci. Rep..

[21] Yang K., Wang X., Zhang C., Liu D., Tao L. (2024). Metformin improves HPRT-targeted purine metabolism and repairs NR4A1-mediated autophagic flux by modulating FoxO1 nu-cleocytoplasmic shuttling to treat postmenopausal osteoporosis. Cell Death Dis..

[22] Liu D., Jin X., Yu G., Wang M., Liu L., Zhang W., Wu J., Wang F., Yang J., Luo Q. (2021). Oleanolic acid blocks the purine salvage pathway for cancer therapy by inactivating SOD1 and stimulating lysosomal proteolysis. Mol. Ther. Oncolytics.

[23] Wang Y., Deng M., Deng B., Ye L., Fei X., Huang Z. (2019). Study on the diagnosis of gout with xanthine and hypoxanthine. J. Clin. Lab. Anal..

[24] Sekine M., Okamoto K., Pai E.F., Nagata K., Ichida K., Hille R., Nishino T. (2023). Allopurinol and oxypurinol differ in their strength and mechanisms of inhibition of xanthine oxidoreductase. J. Biol. Chem.

